# A Novel Graph Constructor for Semisupervised Discriminant Analysis: Combined Low-Rank and *k*-Nearest Neighbor Graph

**DOI:** 10.1155/2017/9290230

**Published:** 2017-02-20

**Authors:** Baokai Zu, Kewen Xia, Yongke Pan, Wenjia Niu

**Affiliations:** ^1^School of Electronic and Information Engineering, Hebei University of Technology, Tianjin 300401, China; ^2^Key Lab of Big Data Computation of Hebei Province, Tianjin 300401, China; ^3^Computer Science Department, Worcester Polytechnic Institute, Worcester, MA 01609, USA

## Abstract

Semisupervised Discriminant Analysis (SDA) is a semisupervised dimensionality reduction algorithm, which can easily resolve the out-of-sample problem. Relative works usually focus on the geometric relationships of data points, which are not obvious, to enhance the performance of SDA. Different from these relative works, the regularized graph construction is researched here, which is important in the graph-based semisupervised learning methods. In this paper, we propose a novel graph for Semisupervised Discriminant Analysis, which is called combined low-rank and *k*-nearest neighbor (LRKNN) graph. In our LRKNN graph, we map the data to the LR feature space and then the *k*NN is adopted to satisfy the algorithmic requirements of SDA. Since the low-rank representation can capture the global structure and the *k*-nearest neighbor algorithm can maximally preserve the local geometrical structure of the data, the LRKNN graph can significantly improve the performance of SDA. Extensive experiments on several real-world databases show that the proposed LRKNN graph is an efficient graph constructor, which can largely outperform other commonly used baselines.

## 1. Introduction

For the real-world data mining and pattern recognition applications, the labeled data are very expensive or difficult to obtain, while the unlabeled data are often copious and available. So how to improve the learning performance using the copious unlabeled data has attracted considerable attention [1, 2]. Semisupervised dimensionality reduction can be directly used in the whole dataset which does not need training set and testing set [3].

Illuminated by semisupervised learning [4–6], Semisupervised Discriminant Analysis (SDA) is first proposed by Cai et al. [2]. It can easily resolve the out-of-sample problem [7]. In SDA algorithm, the labeled samples are used to maximize the different classes reparability and the unlabeled ones to estimate the data's intrinsic geometric information. From then on, many kinds of semisupervised LDA were proposed. Zhang and Yeung proposed SSDA [3] using path-based similarity measure. In a similar way, SMDA [8] and UDA [9] execute LDA under semisupervised setting manifold regularization. And [6] utilizes unlabeled data to maximize an optimality criterion of LDA and uses the constrained concave-convex procedure to solve the optimization problem and so forth.

Although these methods perform semisupervised LDA in different ways, they all need the geometric relationships between the whole data by constructing a regularized graph. The graph remarkably impacts the performance of these methods. However, little attention has been paid to graph constructor methods. So in this paper we study the regularized graph construct problem of SDA [2]. Below we summarize our main contributions in this paper.

- Inspired by low-rank representation (LRR) [10] and the *k*-nearest neighbor algorithm, we construct a novel graph called combined low-rank and *k*-nearest neighbor graph. LRR jointly obtains the representation of all the samples under a global low-rank constraint. Thus it is better at capturing the global data structures.
- Since *k*NN is used to satisfy the algorithmic requirements of SDA, the affinity of local geometrical structure can be maximally preserved after using the LRKNN graph.
- Extensive experiments on real-world datasets show that our proposed LRKNN regularized graph can significantly boost the performance of Semisupervised Discriminant Analysis.

The rest of the paper is organized as follows. We briefly review the related work in Section 2. We give the preliminary in Section 3. We then introduce the combined low-rank and *k*-nearest neighbor graph construct framework in Section 4. Then Section 5 reports the experiment results on real-world database tasks. In Section 6, we conclude the paper.

## 2. Related Work

This paper proposes a combined low-rank and *k*-nearest neighbor graph to boost the performance of Semisupervised Discriminant Analysis. Our work is related to both Semisupervised Discriminant Analysis improvement techniques and graph conductor design. We briefly discuss both of them.

Cai et al. [2] proposed a semisupervised dimensionality reduction algorithm SDA, which captures the local structure for data dimensionality reduction. Zhang and Yeung proposed SSDA [3] using path-based similarity measure to capture global manifold structure of the data. The works in SMDA [8] and UDA [9] also perform semisupervised LDA with manifold regularization. Nie et al. [11] proposed an orthogonal constraint semisupervised orthogonal discriminant analysis method. Zhang et al. [1] utilized must-link constraints or cannot-link constraints to capture the underlying structure of dataset. Song et al. [5] utilized labeled data to discover class structure and utilized unlabeled data to capture the intrinsic local geometry. Probabilistic Semisupervised Discriminant Analysis (PSDA) algorithm is presented by Li et al. [12], which utilizes unlabeled samples to approximate class structure instead of local geometry. In the work [13], Dhamecha et al. presented an incremental Semisupervised Discriminant Analysis algorithm, which utilizes the unlabeled data for enabling incremental learning. The work [14] developed a graph-based semisupervised learning method based on PSDA for dimensionality reduction.

Our work is also related to another line of research, the graph conductor design. There are many methods proposed for graph construction, including *k*-nearest neighbors based method and *ε*-ball based method [15] which are two most popular methods for graph adjacency construction. Based on these two methods, various approaches such as heat kernel [15] and inverse Euclidean distance [16] are used to set the graph edge weights. However, all these methods are to find pairwise Euclidean distances, which are very sensitive to data noise. Moreover, since only the local pairwise relationship between data points is taken into account, the constructed graph cannot reveal sufficiently the clustering relationship among the samples. Yan et al. proposed an *l*_1_-graph via sparse representation [10, 17]. An *l*_1_-graph over a dataset is derived by encoding each datum as a sparse representation of the remaining samples. In the work [18], Zhuang et al. proposed a novel method to construct an informative low-rank graph (LR-graph) for semisupervised learning. And Gao et al. proposed a novel graph construction method via group sparsity [19]. Li and Fu [20] developed an approach to construct graph-based on low-rank coding and *b*-matching constraint and proposed a novel supervised regularization based robust subspace (SRRS) approach via low-rank learning [21]. Zhao et al. proposed a novel approach to construct a sparse graph with blockwise constraint for face representation, named SGB [22]. A sparse and low-rank graph-based discriminant analysis (SLGDA) is proposed, which combines both sparsity and low rankness to maintain global and local structures simultaneously [23]. In the work [24], Li and Fu incorporated KNN constraint and *b*-matching constraint into the low-rank representation model as the balanced (or unbalanced) graph. We focus on constructing a novel graph for SDA, capturing the data using LRR and then utilizing the KNN algorithm to satisfy the algorithmic requirements of SDA.

The work that is most closely related to ours is the low-rank kernel-based Semisupervised Discriminant Analysis [25], which is my previous research. The LRR is used as the kernel in the KSDA [2]. In our current work, we proposed a novel graph for Semisupervised Discriminant Analysis, which is called combined low-rank and *k*-nearest neighbor (LRKNN) graph. In our LRKNN graph, the *k*NN is adopted to satisfy the algorithmic requirements of SDA. Since the low-rank representation can capture the global structure and the *k*-nearest neighbor algorithm can maximally preserve the local geometrical structure of the data, therefore the LRKNN graph can capture not only the global structure but also the local information of the data, which can largely improve the performance of the SDA.

## 3. Preliminary

### 3.1. Overview of SDA

Given a set of samples [**x**_1_,…, **x**_*m*_, **x**_*m*+1_,…, **x**_*m*+*l*_], where *N* = *m* + *l*, the first *m* samples are labeled [**y**_1_,…, **y**_*m*_], and the remaining *l* are unlabeled ones. They all belong to *c* classes. The SDA method [2] hopes to find a rejection matrix **a**, which motivates presenting the prior assumption of consistency by a regularized term. The objective function is as follows:

(1) $$\begin{array}{c} a=\underset{a}{arg\;max}\frac{a^{T}S_{b}a}{a^{T}S_{t}a+\alpha J\left(a\right)}, \end{array}$$

where **S**_*b*_ and **S**_*t*_ are the between-class scatter and total class scatter matrix. And **S**_*w*_ is defined as the within-class scatter matrix.

The parameter *α* in (1) balances the model complexity and the empirical loss. The regularized term supplies us with the flexibility to incorporate the prior knowledge in the applications. We aim at constructing *J*(**a**) graph combining the manifold structure through the available unlabeled samples.

Given a set of samples {**x**_*i*_}_*i*=1_^*m*^, we can construct the graph **G** to represent the relationship between nearby samples by *k*NN. Then put an edge between *k*-nearest neighbors of each other. The corresponding weight matrix **S** is defined as follows:

(2) $$\begin{array}{c} S_{ij}=\left\{\begin{array}{cc} 1, & \text{if }x_{i}\in N_{k}\left(x_{j}\right)\text{ or }x_{j}\in N_{k}\left(x_{i}\right)\\ 0, & \text{otherwise}, \end{array}\right. \end{array}$$

where *N*_*k*_(**x**_*i*_) denotes the set of *k*-nearest neighbors of **x**_*i*_. Then *J*(**a**) term can be defined as follows:

(3) Ja∑ijaTxi−aTxj2Sij=2∑iaTxiDiixiTa−2∑ijaTxiSijxjTa=2aTXD−SXTa=2aTXLXTa,

where **D** is a diagonal matrix whose entries are column (or row since **S** is symmetric) sum of **S**; that is, **D**_*ii*_ = ∑_*j*_**S**_*ij*_. The Laplacian matrix [10] is **L** = **D** − **S**. We can get the objective function of the SDA with the regularizer term *J*(**a**):

(4) $$\begin{array}{c} a=\underset{a}{max}\frac{a^{T}S_{b}a}{a^{T}\left(S_{t}+\alpha XLX^{T}\right)a}. \end{array}$$

By maximizing the generalized eigenvalue problem, we can obtain the projective vector **a**.

(5) $$\begin{array}{c} S_{b}a=\lambda \left(\;S_{t}+\alpha XLX^{T}\;\right)a. \end{array}$$

### 3.2. Low-Rank Representation

 Yan and Wang proposed the low-rank representation and used it to construct the affinities of an undirected graph (here called LR-graph) [10]. It jointly obtains the representation of all the samples under a global low-rank constraint, and thus it is better at capturing the global data structures [16].

Let **X** = [**x**_1_, **x**_2_,…, **x**_*n*_] be a set of samples; each column is a sample which can be represented by a linear combination in the dictionary **A** [26]. Here, we select the samples themselves **X** as the dictionary **A**:

(6) $$\begin{array}{c} X=AZ, \end{array}$$

where **Z** = [**z**_1_, **z**_2_,…, **z**_*n*_] is the coefficient matrix with each **z**_*i*_ being the representation coefficient of **x**_*i*_. LRR seeks the lowest rank solution by solving the following optimization problem [26]:

(7) minZ rankZs.t. X=AZ.

The above optimization problem can be relaxed to the following convex optimization [27]:

(8) minZ Z∗s.t. X=AZ.

Here, ‖·‖_*∗*_ denotes the nuclear norm (or trace norm) [28] of a matrix, that is, the sum of the matrixes singular values. By considering the noise or corruption in our real-world applications, a more reasonable objective function is

(9) minZ,E Z∗+λEls.t. X=AZ+E,

where ‖·‖_*l*_ can be the *l*_2,1_-norm or *l*_1_-norm. In this paper we choose *l*_2,1_-norm as the error term measurement which is defined as ${\left\|E\right\|}_{2,1}=\sum_{j=1}^{n}\sqrt{\sum_{i=1}^{n}{\left({\left[E\right]}_{ij}\right)}^{2}}$. The parameter *λ* is used to balance the effect of low rank and the error term. The optimal solution **Z**^*∗*^ can be obtained via the inexact augmented Lagrange multipliers (ALM) method [29, 30].

### 3.3. *k*-Nearest Neighbor Algorithm

The samples **x**_*i*_ and **x**_*j*_ are considered as neighbors if **x**_*i*_ is among the *k*-nearest neighbors of **x**_*j*_ or **x**_*j*_ is among the *k*-nearest neighbors of **x**_*i*_. There are different methods to assign weights for **W**. The following are three of them.

(i) Inverse of Euclidean distance [16] (here we call it KNNE to distinguish different ones):
  (10) Wij=xi−xj−2,if  xi∈Nkxj  or  xj∈Nkxi0,otherwise.
(ii) 0-1 weighting [15] (here we call it KNNB), where it is used in the original SDA:
  (11) $$\begin{array}{c} W_{ij}=\left\{\begin{array}{cc} 1, & \text{if }x_{i}\in N_{k}\left(x_{j}\right)\text{ or }x_{j}\in N_{k}\left(x_{i}\right)\\ 0, & \text{otherwise}. \end{array}\right. \end{array}$$
(iii) Heat kernel weighting [15] (here we call it KNNK):
  (12) Wij=exp−xi−xj22σ2,if  xi∈Nkxj  or  xj∈Nkxi0,otherwise,
where *N*_*k*_(**x**_*i*_) denotes *k* neighbor neighbors of **x**_*i*_ in (10), (11), and (12). Using this regularization (12), the affinity of local geometrical structure can be maximally preserved.

## 4. Proposed Algorithm

### 4.1. Combined Low-Rank and *k*-Nearest Neighbor (LRKNN) Graph Constructor Algorithm

How to find an appropriate subspace for classification is an important task, which we called dimensionality reduction. The dimensionality reduction is aimed at finding labeling of the graph, which is consistent with both the initial labeling and the data's geometry structure (edges and weights **W**).

These proposed SDA methods always analyze the relationship of the data using the mode one-to-others. For example, the most common *k*-nearest neighbor graph only shows the edges and the weight graph should be 1, or the *lle*-graph and the *l*_1_-graph (SR-graph) determine the graph structure weights by the limitation of *l*_2_-norm or the *l*_1_-norm. And the *l*_1_-graphs lack global constraints, which greatly reduce the performance when the data is grossly corrupted. To solve this drawback, Liu et al. proposed the low-rank representation and used it to construct the affinities of an undirected LR-graph [26]. LR-graph jointly obtains the representation of all the samples under a global low-rank constraint, and thus it is better at capturing the global data structures [31].

Since the LR-graph, *l*_1_-graph, and *lle*-graph are asymmetric matrix, in order to satisfy the algorithmic requirements of SDA, similar graph symmetrization process was often used in the previous works; that is, **W**′ = **W** + **W**^*T*^. Since the LRR is good at capturing the global data structures and the local geometrical structure can be maximally preserved by the *k*-nearest neighbor algorithm, here, we proposed a novel solution which uses *k*-nearest neighbor algorithm to satisfy the algorithmic requirements. So the combined LRKNN method can improve the performance to a very large extent. Heat kernel weighting [15] is used here.

### 4.2. SDA Using Combined Low-Rank and *k*-Nearest Neighbor Graph

Graph structure remarkably impacts the performance of these SDA-likely methods. However, little attention has been paid to graph constructor methods. So in this paper we present a novel combined low-rank and *k*-nearest neighbor graph algorithm, which largely improves the performance of SDA.

Firstly, map the labeled and unlabeled data to the LR-graph feature space. Secondly, obtain the symmetric graph by *k*-nearest neighbor algorithm where heat kernel weighting is used. By choosing appropriate kernel parameter, it can increase the similarities among the intraclass samples and the differences among the interclass samples. Then implement the SDA algorithm for dimensionality reduction. Finally execute the nearest neighbor method for the final classification in the derived low dimensional feature subspace. The procedure is described as follows in Algorithm 1.

## 5. Experiments and Analysis

To examine the performance of the LRKNN graph in SDA algorithm, we conducted extensive experiments on several real-world datasets. In this section, we introduce the datasets we used and the experiments we performed, respectively; then we present the experimental results as well as the analysis. The experiments are conducted on machines with Intel Core CPUs of 2.60 GHz and 8 GB RAM.

### 5.1. Experiment Overview

#### 5.1.1. Datasets

We evaluate the proposed method on 4 real-world datasets including three face databases and the USPS database. In these experiments, we normalize the sample to a unit norm.

*(i) ORL Database [10]*. The ORL dataset contains 10 different images of each for 40 distinct subjects. The images are taken at different times, varying the lighting, facial expressions, and facial details. Each face image is manually cropped and resized to 32 × 32 pixels, with 256 grey levels per pixel.

*(ii) Extended Yale Face Database B [32]*. This dataset now has 38 individuals and around 64 near frontal images under different illuminations per individual. Each face image is resized to 32 × 32 pixels. And we select the first 20 persons and choose 20 samples of each subject.

*(iii) CMU PIE Face Database [2]*. It contains 68 subjects with 41,368 face images. The face images were captured under varying poses, illuminations, and expressions. The size of each image is resized to 32 × 32 pixels. We select the first 20 persons and choose 20 samples of per subject.

*(iv) USPS Database [33]*. The USPS handwritten digit database is a popular subset containing 9298, 16 × 16 handwritten digit images in total. Here, we randomly select 300 examples for the experiments.

#### 5.1.2. Comparative Algorithms

In order to demonstrate how the SDA dimensionality reduction performance can be improved by the combined LRKNN graph, we list out several graphs also including combined SR and LLE with the KNNK algorithm and the separate algorithm (without *k*NN) SR, LLE, and the KNNK for comparison. For the separate LR, SR, and LLE algorithm, the previous symmetrization process **W**′ = **W** + **W**^*T*^ is used here to satisfy the algorithmic requirements of SDA, which is used in previous works.

*(i) SR-Graph [29]*. SR-graph considers the reconstruction coefficients in the sparse representation by solving the following problem: $\hat{a}={arg\;min}_{a}{\left\|y-Xa\right\|}_{1}$. The graph weight is defined as **W**_*ij*_ = |*a*_*j*_^*i*^|.

*(ii) LLE-Graph [34]*. LLE-graph considers the situation of reconstructing a sample from its neighbor points and then minimizes the *l*_2_ reconstruction error.

(13) min ∑ixi−∑jWijxj2,s.t. ∑jWij=1.

**W**_*ij*_ = 0 if **x**_*j*_ does not belong to the neighbors of **x**_*i*_. The number of the nearest neighbors is set to 4.

*(iii) KNNK Graph [29]*. We adopt Euclidean distance as our similarity measure and use a Gaussian kernel to reweight the edges. The number of the nearest neighbors is set to 4. Similarly, the original SDA using KNNB graph is also set to 4.

### 5.2. Experiment 1: Performances of SDA Using Different Regularized Graphs

**Figure 1:**
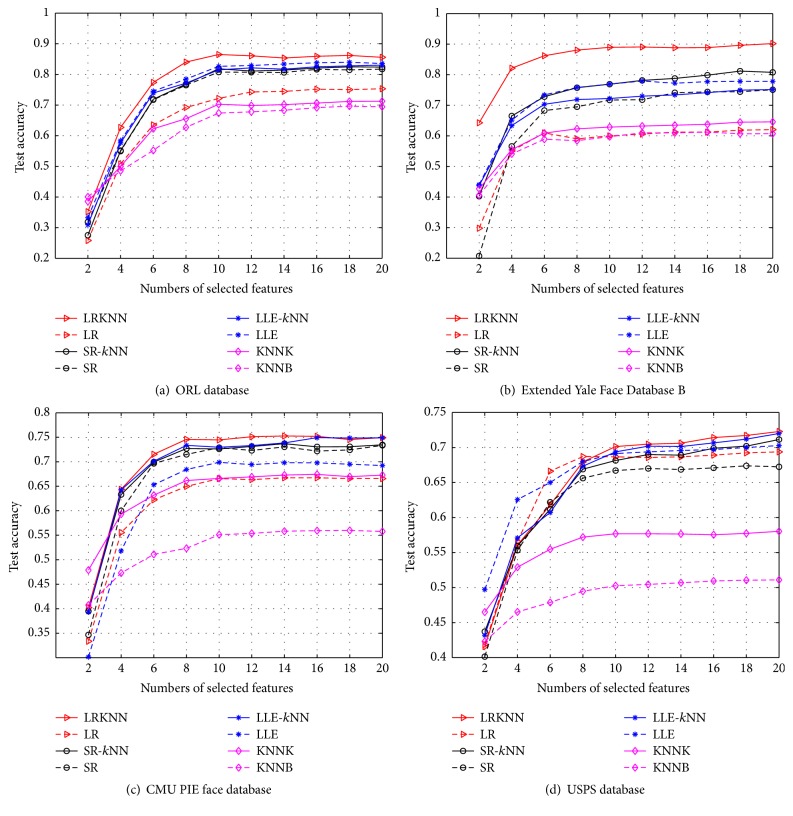
Classification accuracy of different graphs with different selected features.

To examine the effectiveness of the proposed combined LRKNN graph for SDA, we conduct experiments on the four databases. In our experiments, we randomly select 30% samples from each class as the labeled samples to evaluate the performance with different numbers of selected features. The evaluations are conducted with 20 independent runs for each algorithm. We average them as the final results. First we utilize different graph construction methods to get the *J*(**a**) term, and then we implement the SDA algorithm for dimensionality reduction. Finally, the nearest neighbor approach is employed for the final classification in the derived low dimensional feature subspace. For each database, the classification accuracy for different graphs is shown in Figure 1.  Table 1 shows the performance comparison of different graph algorithms. Note that the results are the best results of all these different selected features mentioned above. The bold numbers represent the best results of different graph algorithms. From these results, we can observe the following:

- In most cases, our proposed LRKNN graph consistently achieves the highest classification accuracy compared to the other graphs. The results indicate that the classification accuracy is much higher than the other graph algorithms. So it improves the classification performance to a large extent, which suggests that LRKNN graph is more informative and suitable for SDA.
- In most conditions, the performance of the combined *k*NN algorithm is always superior to the separate algorithm (without *k*NN), which means that our proposed graph construct methods combined *k*NN algorithm is extremely effective, especially for the LRR algorithm.
- Since the SR-graph (*l*_1_-graph) lacks global constraints, the performance improvement is not obvious even if it is combined with the *k*NN algorithm.
- In some cases (maybe some certain enough high dimensionality), the traditional construct graph methods such as *k*NN-graph and LLE-graph may achieve good performances in some databases, but they are not as stable as our proposed algorithm.

Table 2 shows the execution time of the eight methods mentioned. We compute the total time with 20 independent runs for 10 features. And Table 2 gives the average runtime of the 20 runs for 10 features. We can see that although our algorithm is slower than the traditional *k*NN algorithms, the performance is much better than these baseline algorithms at an acceptable runtime.

### 5.3. Experiment 2: Parameters Settings

**Figure 2:**
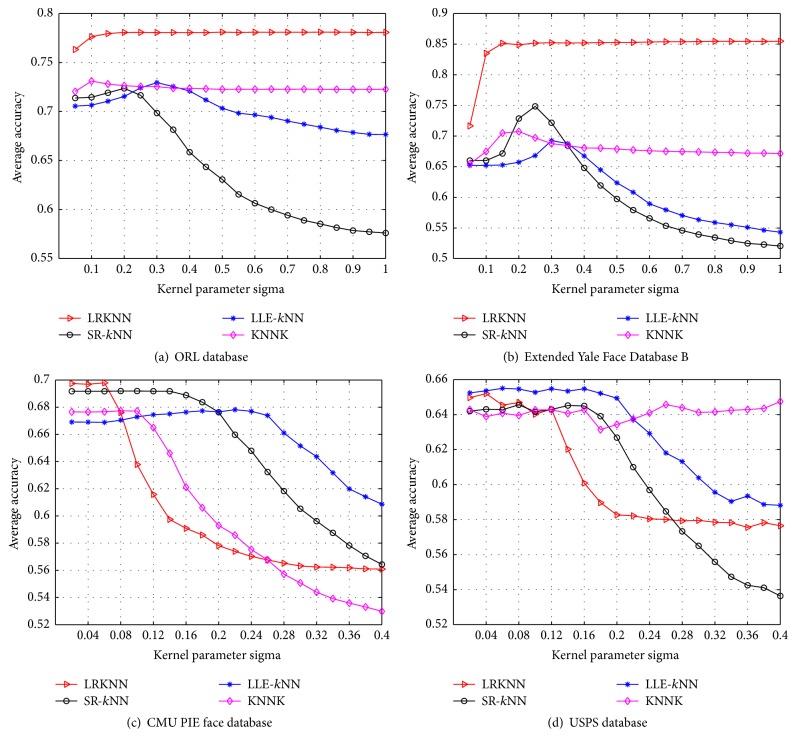
Classification accuracy of different graphs with varying kernel parameters *σ*.

We examine the effect of the heat kernel parameters *σ* in LRKNN, SR-*k*NN, LLE-*k*NN, and KNNK graph. We vary the graph parameters *σ* and examine the classification accuracy on the four databases. We also select 30% samples from each class to evaluate the classification performance. The evaluations are conducted with 20 independent runs and the averaged results are adopted. We adopt the average results of the 10 different numbers of selected features mentioned in Section 5.2 as the final result, which are shown in Figure 2. We can see that the classification accuracy is influenced by the kernel parameters.

**Figure 3:**
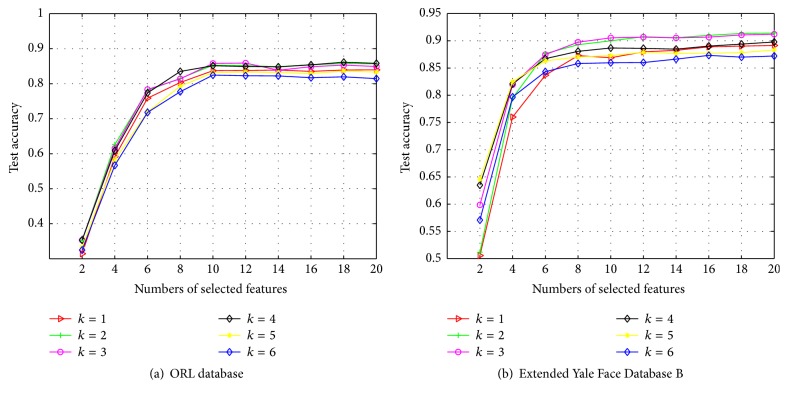
Classification accuracy of LRKNN with nearest neighbor numbers *k*.

We also evaluate the performance of different nearest neighbor numbers for the LRKNN graph, namely, the value *k* for the *k*NN algorithm. Here we conduct the experiments on the ORL database and Extended Yale Face Database B. The procedure is the same as the experiments above. We adopt the average results of the 20 different runs as the final result, which are shown in Figure 3. We can see that the classification accuracy is improving by the increasing of numbers of nearest neighbors. And when the nearest neighbors reach some numbers like 3 or 4, the performance has a slight decrease, since here we choose 4 as the number of nearest neighbors in our experiments.

### 5.4. Experiment 3: Influence of the Label Number

We evaluate the influence of the label number in this subsection. The experiments are conducted with 20 independent runs for each algorithm. We average them as the final results. The procedure is the same as the experiments in Section 5.2. The bold numbers represent the best results. And the percentage number after the database is the label rate. For each database, we vary the percentage of labeled samples from 20% to 50% and the recognition accuracy is shown in Table 3, from which we observe the following.

In most cases, our proposed LRKNN graph consistently achieves the best results, which is robust to the label percentage variations. And it is worth noting that even in very low label rate our proposed method can achieve high classification accuracy, while some other compared algorithms are not as robust as our LRKNN algorithm especially when the label rate is low. Thus, our proposed method has much superiority compared with the traditional construct graph methods. Sometimes these traditional methods may achieve good performances in some databases with high enough label rate. But they are not as stable as our proposed algorithm. Since the labeled data is very expensive and difficult, our proposed graph for SDA algorithm is more robust and suitable for the real-world data.

### 5.5. Experiment 4: Performance of LRKNN Graph with Different Weight Methods

**Figure 4:**
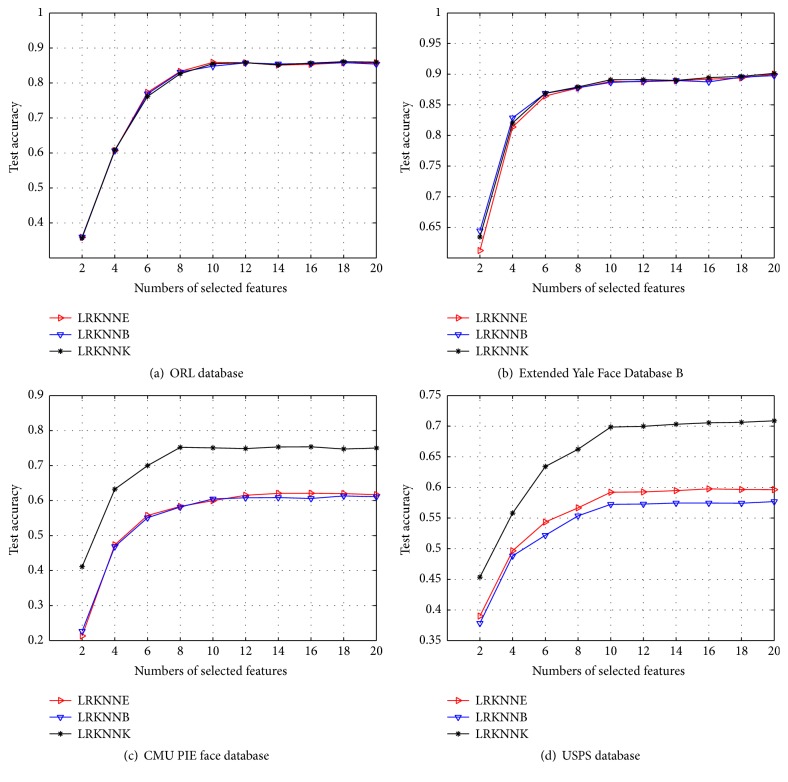
Classification accuracy of three weight methods for LRKNN graph.

We evaluate the performance of the different weight methods mentioned in Section 5.2 for our LRKNN graph. We conduct 20 independent runs for each algorithm. We average them as the final results. The procedure is the same as the experiments in Section 5.2. For each database, we show the performance for the three weight methods (KNNE, KNNB, and KNNK) of *k*NN for our LRKNN graph in Figure 4, from which we observe the following.

Overall, the KNNK based LRKNN graph achieves the best results compared with the other two *k*NN methods. And we can see that in some datasets the performance gap of the three *k*NN methods is very small, while in some other datasets the performance gap is much bigger, since the KNNE and KNNB cannot capture the local structure very well in some datasets. They are not as stable as KNNK algorithm, since here we choose the heat kernel weighting method for LRKNN graph.

### 5.6. Experiment 5: Robustness to Different Types of Noises

In this test we compare the performance of different graphs in the noisy environment. Extended Yale Face Database B is used in this experiment. The Gaussian white noise, “salt and pepper” noise, and multiplicative noise are added to the data, respectively. The Gaussian white noise is with mean 0 and different variances from 0 to 0.1. The “salt and pepper” noise is added to the image with different noise densities from 0 to 0.1. And multiplicative noise is added to the data *I*, using the equation *J* = *I* + *n∗I*, where *I* and *J* are the original and noised data and *n* is uniformly distributed random noise with mean 0 and varying variance from 0 to 0.1.

The number of labeled samples in each class is 30%. The experiments are conducted 20 runs for each graph. We average them as the final results. The procedure is the same as the experiments in Section 5.2. The bold numbers represent the best results. For each graph, we vary the parameter of different noise. The results are shown in Tables 4, 5, and 6. As we can see, the results of our method are stable for Gaussian noise, “salt and pepper” noise, and multiplicative noise. And because of the robustness of the low-rank representation to noise, our method LRKNN is much more robust than other graphs. With the different kinds of gradually increasing noise, some kinds of methods' performance fall a lot, while our method's performance is robust and decrease little with the increasing noises.

## 6. Conclusions

In this paper, we propose a novel combined low-rank and *k*-nearest neighbor graph algorithm, which largely improves the performance of SDA. The LRR can naturally capture the global structure of the data. And the *k*-nearest neighbor algorithm can maximally preserve the local geometrical structure of the data. Therefore, it can largely improve the performance using the *k*NN algorithm to satisfy the SDA's algorithmic requirements. Empirical studies on four real-world datasets show that our proposed LRKNN graph for Semisupervised Discriminant Analysis is more robust and suitable for the real-world applications.

## Figures and Tables

**Algorithm 1:**
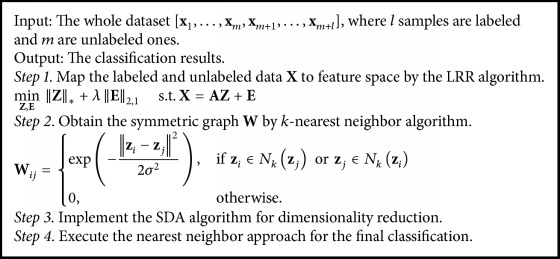
Procedure of SDA using combined low-rank and *k*-nearest neighbor graph.

**Table 1:** Classification accuracy of different methods on real-world databases.

Graphs	LRKNN	LR	SR-*k*NN	SR	LLE-*k*NN	LLE	KNNK	KNNB
ORL	**0.865**	0.753214	0.824643	0.8175	0.830357	0.84	0.712444	0.696483
YaleB	**0.901786**	0.620357	0.811786	0.750714	0.751429	0.778214	0.645828	0.612487
PIE	**0.752692**	0.667308	0.736923	0.733462	0.749231	0.698846	0.674269	0.559724
USPS	**0.722857**	0.69381	0.711429	0.67381	0.72	0.702857	0.580313	0.510963

**Table 2:** Run time of different methods on real-world databases (unit (s)).

Graphs	LRKNN	LR	SR-*k*NN	SR	LLE-*k*NN	LLE	KNNK	KNNB
ORL	17.5078083	17.16502055	18.81038385	18.745907	2.20949085	2.1973647	2.1734913	2.1167409
YaleB	17.0695632	16.6934086	18.54172935	18.2687115	1.80366055	1.75954255	1.7359231	1.70582745
PIE	16.77245375	16.61093775	18.33027985	18.4719838	1.9091679	1.8791345	1.8077919	1.80170705
USPS	6.0444118	6.1392268	4.1187905	3.94524935	1.12164545	1.11113225	1.12937165	1.1236474

**Table 3:** Classification accuracy of different graphs with different label rates on four databases.

Graphs	LRKNN	LR	SR-*k*NN	SR	LLE-*k*NN	LLE	KNNK	KNNB
ORL (20%)	**0.739063**	0.564375	0.695	0.659688	0.675938	0.694063	0.438489	0.432261
ORL (30%)	**0.865**	0.753214	0.824643	0.8175	0.830357	0.84	0.696483	0.712444
ORL (40%)	**0.9275**	0.8575	0.9075	0.915417	0.916667	0.917083	0.889171	0.880085
ORL (50%)	**0.9615**	0.929	0.9535	0.959	0.953	0.944893	0.941861	0.9615
YaleB (20%)	**0.872188**	0.436875	0.704063	0.613438	0.645	0.684688	0.421005	0.435515
YaleB (30%)	**0.901786**	0.620357	0.811786	0.750714	0.751429	0.779286	0.612487	0.645828
YaleB (40%)	**0.915833**	0.742917	0.875417	0.840833	0.832083	0.829167	0.741223	0.789611
YaleB (50%)	**0.9385**	0.7805	0.931	0.892	0.8835	0.8925	0.824813	0.902639
PIE (20%)	**0.623333**	0.521667	0.622333	0.594667	0.594667	0.578667	0.376174	0.464566
PIE (30%)	**0.752692**	0.667308	0.736923	0.733462	0.749231	0.698846	0.559724	0.674269
PIE (40%)	0.820909	0.761364	0.839091	0.839091	**0.852273**	0.815455	0.72197	0.846924
PIE (50%)	0.885	0.833889	0.888333	0.881667	0.867222	**0.908269**	0.855231	0.875
USPS (20%)	**0.685**	0.684583	0.664167	0.685417	0.675417	0.609583	0.389417	0.41954
USPS (30%)	**0.722857**	0.69381	0.711429	0.67381	0.72	0.702857	0.510963	0.580313
USPS (40%)	**0.793667**	0.788333	0.787778	0.793333	0.790556	0.78	0.744394	0.765773
USPS (50%)	0.830667	0.844667	0.835333	0.828	0.827333	0.849333	**0.86419**	0.862344

**Table 4:** Classification accuracy of different graphs with varying variance Gaussian noise.

Gaussian	0	0.02	0.04	0.06	0.08	0.1
LRKNN	**0.901786**	**0.856786**	**0.845**	**0.851071**	**0.841071**	**0.826786**
LR	0.620357	0.544643	0.543214	0.552857	0.543929	0.535357
SR-*k*NN	0.811786	0.785714	0.799286	0.788214	0.780357	0.776786
SR	0.750714	0.558571	0.572857	0.588214	0.621429	0.629643
LLE-*k*NN	0.751429	0.741071	0.724643	0.735	0.726429	0.727857
LLE	0.779286	0.725	0.713214	0.713214	0.717143	0.716429
KNNK	0.612487	0.547453	0.542545	0.548299	0.553657	0.556745
KNNB	0.645828	0.579001	0.571481	0.573906	0.563472	0.575386

**Table 5:** Classification accuracy of different graphs with varying densities “salt and pepper” noise.

“Salt and pepper”	0	0.02	0.04	0.06	0.08	0.1
LRKNN	**0.901786**	**0.881786**	**0.842857**	**0.831786**	**0.799286**	**0.760714**
LR	0.620357	0.559643	0.524286	0.505	0.493214	0.47
SR-*k*NN	0.811786	0.803571	0.778571	0.755357	0.735714	0.710714
SR	0.750714	0.738214	0.680357	0.6425	0.617143	0.61285
LLE-*k*NN	0.751429	0.732857	0.655357	0.559286	0.496071	0.455357
LLE	0.779286	0.737143	0.670714	0.569286	0.508571	0.472143
KNNK	0.612487	0.551039	0.504207	0.466663	0.442531	0.43228
KNNB	0.645828	0.586118	0.536622	0.498804	0.484881	0.468962

**Table 6:** Classification accuracy of different graphs with varying variance multiplicative noise.

Multiplicative	0	0.02	0.04	0.06	0.08	0.1
LRKNN	**0.901786**	**0.883571**	**0.886071**	**0.881786**	**0.873214**	**0.855357**
LR	0.620357	0.597143	0.579643	0.566786	0.545	0.536429
SR-*k*NN	0.811786	0.804286	0.779286	0.750357	0.75	0.719286
SR	0.750714	0.7225	0.657857	0.574286	0.52	0.47
LLE-*k*NN	0.751429	0.731071	0.645357	0.548214	0.510714	0.447857
LLE	0.779286	0.724643	0.658214	0.566786	0.507857	0.472143
KNNK	0.612487	0.551147	0.508219	0.466734	0.4441	0.423919
KNNB	0.645828	0.587767	0.528635	0.498222	0.481003	0.476331
